# Platelet-Rich Plasma-Loaded Poly(d,l-lactide)-Poly(ethylene glycol)-Poly(d,l-lactide) Hydrogel Dressing Promotes Full-Thickness Skin Wound Healing in a Rodent Model

**DOI:** 10.3390/ijms17071001

**Published:** 2016-06-24

**Authors:** Manle Qiu, Daoyun Chen, Chaoyong Shen, Ji Shen, Huakun Zhao, Yaohua He

**Affiliations:** 1 Department of Sports Medicine, Shanghai Jiao Tong University Affiliated Sixth People’s Hospital, 600 Yishan Road, Shanghai 200233, China; qml198816@aliyun.com (M.Q.); dychen1218@163.com (D.C.); shenjidoctor@163.com (J.S.); zhkmssj@126.com (H.Z.); 2 Department of Gastrointestinal Surgery, West China Hospital, Sichuan University, Chengdu 610041, Sichuan, China; scyshenchaoyong@163.com

**Keywords:** PDLLA-PEG-PDLLA, PLEL hydrogel, PRP, drug-delivery system, wound healing, angiogenesis

## Abstract

Traditional therapeutic methods for skin wounds have many disadvantages, and new wound dressings that can facilitate the healing process are thus urgently needed. Platelet-rich plasma (PRP) contains multiple growth factors (GFs) and shows a significant capacity to heal soft tissue wounds. However, these GFs have a short half-life and deactivate rapidly; we therefore need a sustained delivery system to overcome this shortcoming. In this study, poly(d,l-lactide)-poly(ethylene glycol)-poly(d,l-lactide) (PDLLA-PEG-PDLLA: PLEL) hydrogel was successfully created as delivery vehicle for PRP GFs and was evaluated systematically. PLEL hydrogel was injectable at room temperature and exhibited a smart thermosensitive in situ gel-formation behavior at body temperature. In vitro cell culture showed PRP-loaded PLEL hydrogel (PRP/PLEL) had little cytotoxicity, and promoted EaHy926 proliferation, migration and tube formation; the factor release assay additionally indicated that PLEL realized the controlled release of PRP GFs for as long as 14 days. When employed to treat rodents’ full-thickness skin defects, PRP/PLEL showed a significantly better ability to raise the number of both newly formed and mature blood vessels compared to the control, PLEL and PRP groups. Furthermore, the PRP/PLEL-treated group displayed faster wound closure, better reepithelialization and collagen formation. Taken together, PRP/PLEL provides a promising strategy for promoting angiogenesis and skin wound healing, which extends the potential of this dressing for clinical application.

## 1. Introduction

Therapy for full-thickness skin wounds remains a clinical challenge because traditional treatments have several problems [1,2,3,4,5]. For example, an autologous skin graft is limited by restricted sources and defects to donor sites, while allograft and xenograft grafts may cause immunologic rejection and infectious disease transmission. Hence, the development of new generation dressings has become a research hot spot. It is generally considered that multiple cell types and cellular signals participate in skin wound healing, mediated by various growth factors (GFs), cytokines and chemokines [6]. Therefore, applications of GFs have attracted increasing research interests to serve as the most straightforward treatment option for skin wounds, since they could directly act on healing-related cells and stimulate the formation of functional vascular networks.

PRP—a concentrated volume of plasma rich in platelets—is an emerging technique widely applied in clinics including orthopedics, sports medicine and dermatologic surgery [7,8,9]. By providing multiple naturally occurring GFs, PRP promotes healing processes of respective tissue types [10,11]. PRP can be collected autologously and rapidly by centrifugation from whole blood, so there is no consideration of antibody formation, contamination or infection [8]. In addition, PRP is cost effective while commercial GF products are prohibitively expensive, and there are always adverse biological effects or even tumor-forming tendencies when recombinant GFs are used at inevitably supra-physiological concentrations [12,13]. In terms of the analysis made above, PRP is likely to be an ideal candidate for enhancing skin wound healing clinically. However, some studies revealed that PRP alone scarcely led to a significant improvement [14], which may be ascribed to the short lifetime of the GFs included or their early inactivation and degradation by numerous hydrolytic enzymes at the wound site [15]. Herein, an ideal delivery vehicle that could make PRP GFs release in a sustained manner is necessary for making optimal use of the PRP method.

Hydrogels have long been studied for their promising application in targeted drug-delivery systems [16]. They have three dimensional interconnected microporous structures similar to that of extracellular matrix (ECM), with high permeability and capacity for water, oxygen, biological fluids, metabolites, and can retain therapeutic agents such as GFs for long-term release [17]. It was reported that topical application of GFs in a hydrogel-based delivery system could promote their therapeutic efficacy in the management of clinical wounds [18]. Various kinds of natural and synthetic polymers have the ability to form hydrogels [19], among them, thermosensitive hydrogels are widely used for tissue regeneration. At ambient temperature or lower, they are in a free-flowing sol state, as soon as applied in vivo, they turn into non-flowing gels in response to body temperature [16]. This unique property makes it possible for us to incorporate pharmaceutical agents into them in the sol state, followed by simply injecting the mixed formulations into target tissue sites to form a stable gel in situ, and finally acting as a sustained drug-release vehicle [20]. Moreover, they can be used as plausible dressings to fill complex-shaped skin wounds without wrinkling or fluting, which is a remarkable advantage over most commercially available preformed materials such as membranes or sheets.

PDLLA and PEG are synthetic polymers, both of them are biocompatible, biodegradable and physicochemically tunable. As a result, they have received special attention in the literature and have already been widely used in several Food and Drug Administration-approved products [21,22,23]. For example, a PLEL-based microcapsule solution was used as a delivery vehicle for BMP-2 and BMP-7 in a bone tissue engineering assay recently, with the results showing a satisfying bone regeneration. In our previous work [20], a novel injectable thermoreversible and thermogelling PLEL hydrogel was developed and showed a significant ability in reducing post-operative adhesion in a sidewall defect-bowel abrasion model in rats. In this study, we pushed ahead its application range. By incorporating PRP suspension in the PLEL aqueous solutions, we successfully prepared a homogeneous PRP/PLEL composite, and evaluated its application prospect to be an ideal wound dressing agent systematically. Firstly, thermosensitive phase transition behavior and biocompatibility of PLEL hydrogel as well as the GF release kinetics of PRP/PLEL were studied in vitro; secondly, the function of PRP/PLEL to heal full-thickness skin wounds was investigated in rodents. 

## 2. Results

### 2.1. Characterization of Poly(d,l-lactide)-Poly(ethylene glycol)-Poly(d,l-lactide) (PLEL) Triblock Copolymers

**Figure 1:**
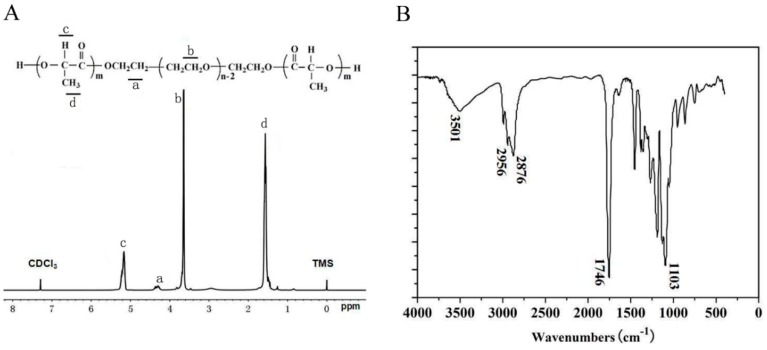
^1^H NMR (**A**) and FTIR (**B**) spectra of PLEL triblock copolymers.

d,l-lactide was subjected to ring-opening copolymerization initiated by PEG using Sn(Oct)_2_ as catalysts to synthesize the PLEL triblock copolymers. In the nuclear magnetic resonance (^1^H NMR, Varian 400 spectrometer, Varian, Santa Clara, CA, USA) spectrum (Figure 1A), peaks at 5.10 ppm and 1.55 ppm were respectively assigned to the protons (–CH–) and methyl protons (–CH_3_) of PDLLA segment. The peak at 4.30 ppm represented the methylene protons (–O–CH_2_–CH_2_–) between d,l-lactide and PEG, and peak at 3.65 ppm corresponded to the methylene protons (–CH_2_–) of PEG [20,24,25]. Furthermore, the macromolecular weight and weight ratios were revealed reasonably close to theoretical value expected on the basis of the feed ratio. In Fourier Transform Infrared (FTIR, 200SXV Infrared Spectrophotometer, Nicolet, Boston, MA, USA) spectrum (Figure 1B), a strong C=O stretching band appeared at 1746 cm^−1^, which was attributed to the ester carbonyl bond. The absorption band at 1103 cm^−1^ was assigned to the representative C–O–C stretching vibrations of repetitive –OCH_2_CH_2_ units in PEG. And the absorption band at 3501 cm^−1^ was attributed to terminal hydroxyl groups (–OH) of the PLEL copolymers [20,26]. These above results confirmed that the as-obtained PLEL triblock copolymers derived from the mentioned copolymerization procedures were synthesized successfully.

### 2.2. Temperature-Dependent Sol–Gel-Precipitation Transition Behavior of PLEL Hydrogel

**Figure 2:**
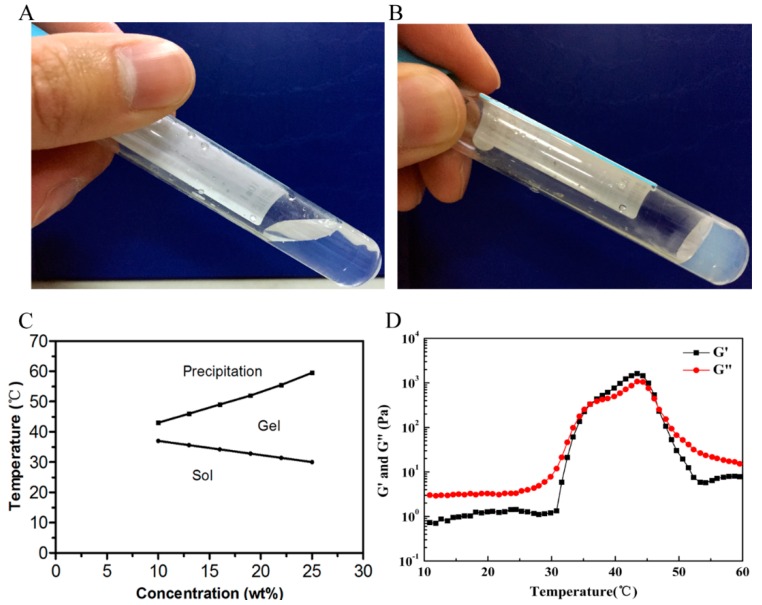
Thermosensitive phase transition behavior and rheological analysis of PLEL hydrogel. (**A**,**B**) Photographs of PLEL hydrogel (25% *w*/*w*) at 20 °C (**A**) and at 37 °C (**B**); (**C**) Sol–gel-precipitation phase transition diagram of PLEL hydrogel at different concentrations; (**D**) Temperature-dependence of storage (*G*’) and loss modulus (*G*”) for PLEL hydrogel (25% *w*/*w*) as a function of temperature.

Figure 2A–C present the notable phase transition diagram of PLEL hydrogel measured by test-tube-inversion method. When PLEL concentration was above a critical gelation concentration (CGC), the phase transition process upon heating was composed of three basic physical states including sol, gel and precipitation. Specifically, sol–gel transition was corresponded to lower critical gelation temperature (LCGT), which seemed to be linked to micelle packing and aggregating [27,28]; gel-precipitation transition relevant to upper critical gelation temperature (UCGT) might be driven by the increased molecular motion of PDLLA block in PLEL hydrogel and collapse of hydrogel microporous structures [28,29]. It has been reported that phase transition behavior of PLEL hydrogel could be tuned by modifying its molecule weight, block length and polymer concentration [20]. Since skin temperature is lower than body core temperature, we chose the concentration of 25% (*w*/*w*) for the following experiments to guarantee its rapid gelation on skin wounds.

To understand how PLEL hydrogel behaves when it is applied on a wound surface, we conducted a dynamic rheological assay to quantitatively observe its sol–gel transition. Here sol–gel transition is defined as the point where storage modulus (*G*’) increases larger than loss modulus (*G*”). It could be observed in Figure 2D that both *G*’ and *G*” of PLEL hydrogel (25% *w*/*w*) were very low (less than 1 Pa) and were essentially independent of temperature from 4 to 30 °C, yet when temperature increased to around body temperature, both G’ and G” raised abruptly more than three magnitudes and *G*’ > *G*” occurred, which led to the sol−gel transition of PLEL hydrogel. These rheological results further prove the injectability of PLEL hydrogel without the risk of syringe clogging upon injection at room temperature, and its in situ gel-forming potential for in vivo applications.

### 2.3. Cytotoxicity Evaluation of PLEL Hydrogel and Its Extract

**Figure 3:**
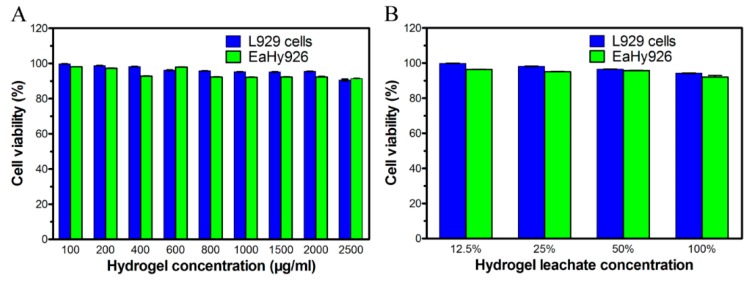
Biocompatibility of PLEL hydrogel and its leachate. Effect of hydrogel in different concentrations (**A**) and the leachate (**B**) on the viability of L929 cells and EaHy926 analyzed with CCK-8 assay. Bars represent mean ± SD (*n* = 5).

The cell toxicity of PLEL hydrogel and hydrogel extractions were evaluated by cell viability assay using murine L929 cells and EaHy926. We chose fibroblasts and vascular endothelial cells here because they exert important function in the process of wound healing, thus the biocompatibility of PLEL and its leaching solutions with these cells were directly related to tissue regeneration potentials. According to Figure 3A, both L929 cells and EaHy926 viability decreased slowly as the PLEL concentration increased, but 85% cell viability was detected even though the concentration was 2500 μg/mL; and the viability of L929 cells or EaHy926 cultured with hydrogel leachate was also nearly 90% (Figure 3B). These results indicated that PLEL hydrogel and its leachate had acceptable cytotoxicity and were safe materials.

### 2.4. Release Kinetics of Platelet-Derived Growth Factor (PDGF)-BB from Platelet-Rich Plasma (PRP) with and without Delivery Vehicle

**Figure 4:**
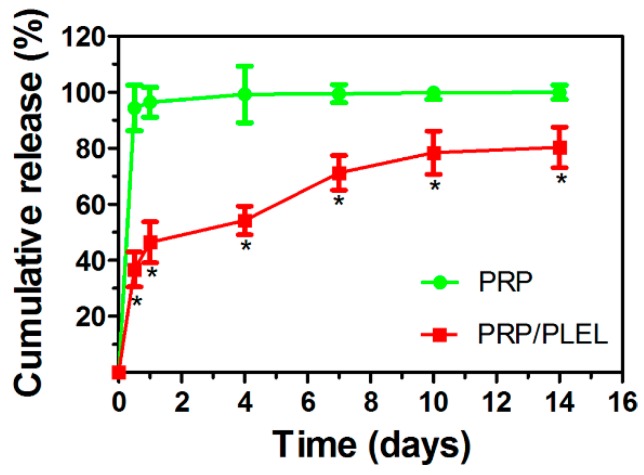
Platelet-derived growth factor-BB (PDGF-BB) release kinetics from PRP and PRP/PLEL in SBF. Data represent mean ± SD (*n* = 5). * *p* < 0.05 between PRP/PLEL and PRP.

Since the amount of platelet-derived growth factor-BB (PDGF-BB) in PRP is relatively high, it was selected as a representative to show the release kinetics of PRP GFs. By using simulated body fluid (SBF) to imitate the body fluid microenvironment, kinetics of PDGF-BB releasing from PRP with and without PLEL hydrogel as a delivery vehicle were compared using enzyme-linked immunosorbent assay (ELISA, R&D Systems, Minneapolis, MN, USA). Release periods differed significantly between PRP alone and PRP/PLEL. As is shown in Figure 4, the initial burst release of PDGF-BB from the PRP group for the first 12 h was 94% ± 8%, while that from PRP/PLEL was 37% ± 6%. PDGF-BB could hardly be detected from the PRP group past the first 4 days, whereas for PRP/PLEL, 80% ± 7% was continuously released over 14 days.

### 2.5. Platelet-Rich Plasma (PRP)/PLEL Promotes Migration and Tube Formation of EaHy926

**Figure 5:**
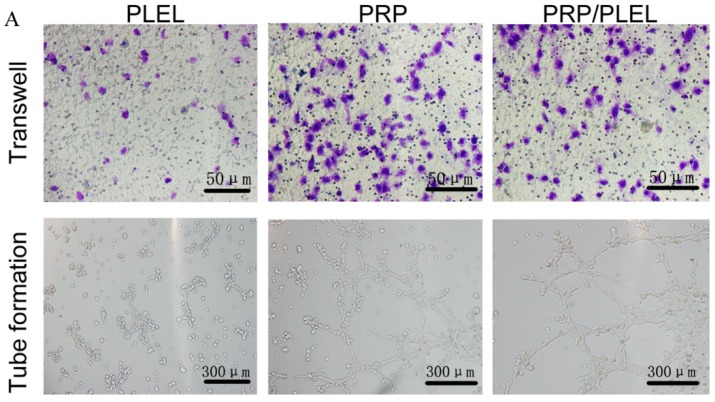

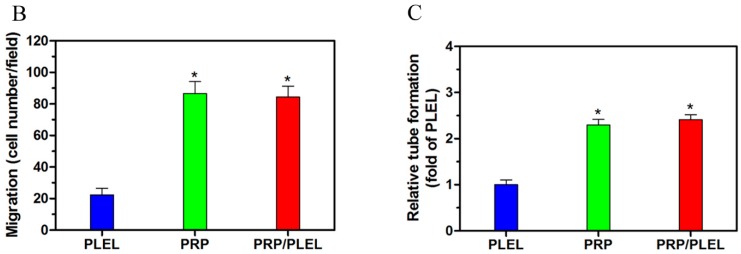
(**A**) Representative photographs showing the positive effect of platelet-rich plasma (PRP) growth factors (GFs) on migration and tube formation of EaHy926 after incubation for 24 h (Scale bar = 50 and 300 μm, respectively); (**B**) Quantitation of EaHy926 migration (blue-stained cells) using a transwell assay; (**C**) Quantitation of tube formation by counting the number of complete capillaries connecting individual points of the polygonal structures. Bars represent mean ± SD (*n* = 5). * *p* < 0.05 compared to PLEL.

As is shown by transwell assay results (Figure 5A,B), after incubation for 24 h, both PRP and PRP/PLEL promoted the migration of EaHy926 significantly when compared to PLEL. Tube-formation analysis (Figure 5A,C) was used to determine the proangiogenic potential of PRP. EaHy926 treated with PRP and PRP/PLEL displayed elongated and tube-like structures on Matrigel (BD Bioscience, San Jose, CA, USA) substratum, whereas cells incubated with PLEL existed in sparse or incomplete tubular networks, which demonstrated that both PRP-involved treatments promoted tube-formation ability of EaHy926. From the above results, we can conclude that PRP indeed involves numerous GFs in bioactive form which facilitate endothelial cell migration and function. 

### 2.6. Bioactivity of Growth Factors (GFs) Released from PRP and PRP/PLEL

**Figure 6:**
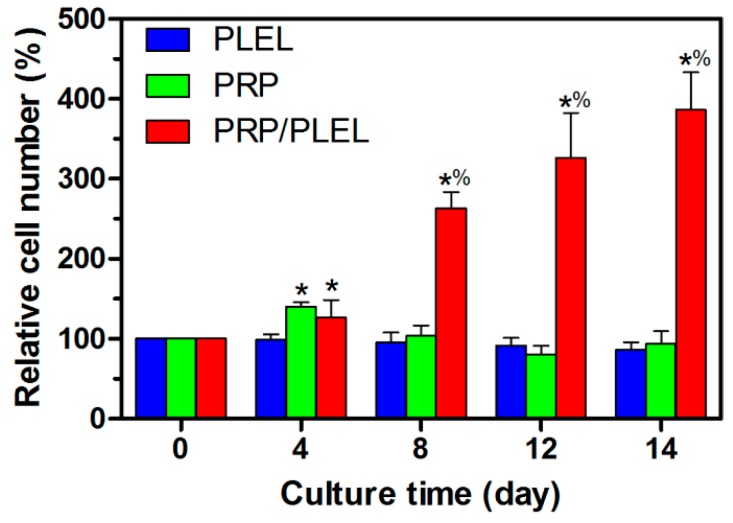
*In vitro* effect of GFs released from PRP and PRP/PLEL on the proliferation of EaHy926. Bars represent mean ± SD (*n* = 5). * *p* < 0.05 compared to PLEL; % *p* < 0.05 compared to PRP.

Observation time for the above transwell assay and tube-formation assay were no longer than 24 h; to confirm our hypothesis that PLEL hydrogel could be a sustained-release vehicle for PRP GFs, however, we need a long-term test. In this part, time interval was set as long as 14 days. The GFs released from PRP and PRP/PLEL showed different bioactivity levels, determined by their capacity to stimulate EaHy926 proliferation. It was revealed in Figure 6 that the PLEL group displayed a slightly decreased number of cells across 14 days, which may be ascribed to the decreased PH accompanying with the generation of d,l-lactide and low-molecular-weight oligomers [20]. Cell number increased significantly in the presence of PRP than PLEL at day 4, while although the cell number in PRP was larger than PRP/PLEL, the difference was not significant. This result was in good accordance with the release kinetics of PRP GFs, especially the initial burst release. That is to say, without a delivery vehicle, PRP GFs can release and exert their efficiency in the first few days after purification; this is also evidenced by a previous report [30]. It was notable that the PRP/PLEL group started to show a significantly promoted cell proliferating tendency from day 8 compared to the other two groups, demonstrating that PLEL hydrogel supported the sustained release of PRP GFs, with their bioactivities reserved. Furthermore, this proliferation profile indicating that the aforementioned acidic effect of PLEL hydrogel during degradation was actually mild, and had little toxic effect on GFs’ bioactivity. The underlying mechanism might be due to the high water capacity in the ECM-mimic hydrogel and rapid diffusion of acidic-degradation products.

### 2.7. Gross Observation of Skin Wounds and Wound Closure Calculation

**Figure 7:**
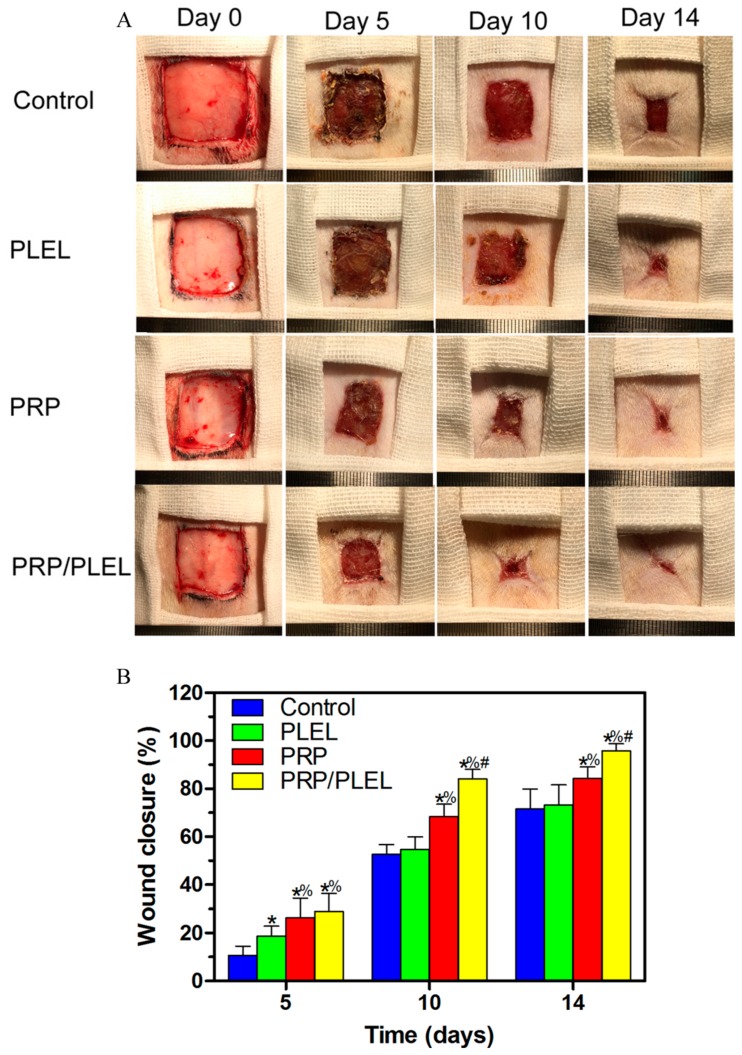
(**A**) Macroscopic full-thickness skin wounds images of control, PLEL, PRP and PRP/PLEL group at 0, 5, 10 and 14 days post surgery; (**B**) Significantly faster wound closure was observed in PRP/PLEL. Bars represent mean ± SD (*n* = 5). * *p* < 0.05 compared to control; % *p* < 0.05 compared to PLEL; # *p* < 0.05 compared to PRP.

All animals showed a good general health condition and there is no sign of infection formation at the wound site; hydrogel-treated wounds remained hydrated throughout the study period. Figure 7A shows typical wound images captured from each group at day 0, 5, 10 and 14. Gross observations revealed increasing wound closure with time in all groups. Particularly, the majority of PRP/PLEL-treated wounds appeared to be healed at day 14, while this phenomenon was not observed in control, PLEL or PRP-treated wounds. According to wound closure calculations (Figure 7B), wounds treated with PRP and PRP/PLEL closed significantly faster than control and PLEL groups at every observation time point. The PRP/PLEL-treated group displayed significantly better wound closure than the PLEL group at time points of day 10 and day 14 post surgery, which provided further evidence that GFs in PRP/PLEL displayed a sustained release form. It was reported that wound closure could be achieved through wound contraction and reepithelialization [31]. Therefore, it was necessary to investigate the effect of PLEL and PRP/PLEL on reepithelialization, which is shown in detail in the next section.

### 2.8. Histologic, Immunohistochemical and Immunofluorescent Evalution

**Figure 8:**
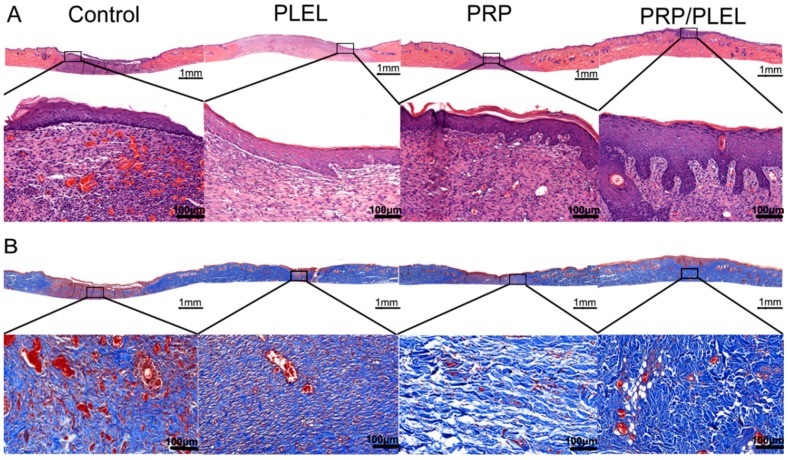
Histological analysis of control, PLEL, PRP and PRP/PLEL group on day 14 post surgery. (**A**) Transmitted light images of HE-stained sections with low magnification (above, scale bar = 1 mm) and high magnification view of the region inside the box (below, scale bar = 100 μm); (**B**) Transmitted light images of Masson’s trichrome-stained sections, with low magnification (scale bar = 1 mm), and an enlarged view of the region inside the box (scale bar = 100 μm).

Assessment of hematoxylin-eosin (HE)-stained slides revealed that 14 days post surgery, PRP/PLEL-treated wounds displayed better reepithelialization compared to control, PLEL or PRP-treated wounds (Figure 8A), and that PRP-treated wounds were better than control or PLEL-treated wounds. Masson’s trichrome staining was employed to demonstrate repair outcomes by visualizing collagen formation. In the sections, collagen and nuclei were marked as blue. After 14 days of surgery, when compared to the other three groups, the PRP/PLEL-treated group exhibited not only significantly higher collagen content, but also these collagen fibers were better organized (Figure 8B).

**Figure 9:**
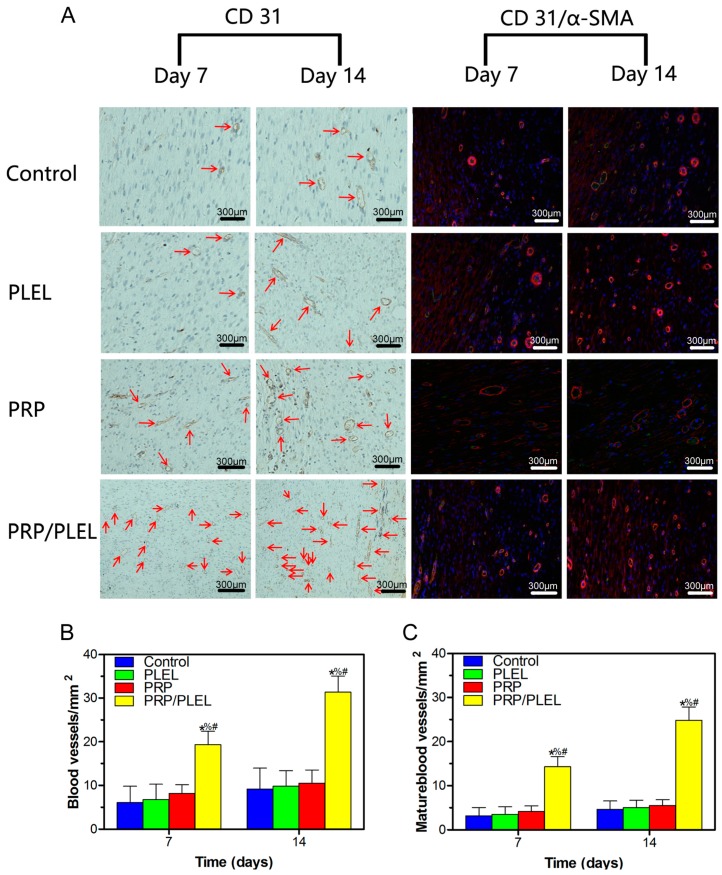
(**A**) Immunohistochemical staining for CD31 (left) and immunofluorescent staining for CD31 and α-SMA (right). Positive CD31 staining was used to show round or oval-shaped newly formed blood vessels (red arrow). Red and green co-staining represents mature blood vessels. Numbers of newly formed blood vessels (**B**) and mature blood vessels (**C**) in control, PLEL PRP and PRP/PLEL group on 7 and 14 days post surgery. Bars represent mean ± SD (*n* = 5). * *p* < 0.05 compared to control; % *p* < 0.05 compared to PLEL; # *p* < 0.05 compared to PRP.

Immunohistochemical staining for CD31 and immunofluorescence staining for CD31 + α-smooth actin (α-SMA) were used to display newly formed and mature blood vessels, respectively. Quantitation was conducted by counting the number of positively stained cells per mm^2^, and it revealed that both of their numbers increased from day 7 to day 14 post surgery in all groups (Figure 9A). For newly formed blood vessels, both at day 7 and 14 after surgery, a significantly higher vascular density existed in the PRP/PLEL-treated group compared to that of the other three groups (Figure 9B); while there was no significant difference among control, PLEL or PRP-treated groups. Similarly, mature blood vessel density presented the same trend as that of the newly formed blood vessels, with the only difference of corresponding lower values (Figure 9C).

## 3. Discussion

The present study demonstrated that the nontoxic PRP/PLEL hydrogel had a capacity to promote angiogenesis and accelerate full-thickness skin wound healing in rodents. In vitro results confirmed our hypothesis that PLEL hydrogel could be an efficient delivery vehicle for PRP GFs with their biological activities maintained.

Wound healing as a dynamic process involves inflammation, proliferation and remodeling phases [6,32]; each stage contains interactions among cells, GFs and ECM. Recently, GFs have become commercially available and are widely used as a novel method in tissue regeneration fields [32], but the results are not always satisfactory. This may partly because a single GF has limitations in inducing optimal biological effects (such as angiogenesis, reepithelialization and collagen deposition), and partly because rapid enzymatic digestion or deactivation of the GFs occur the body fluid environment [14,33]. To solve the problem, different autogenous GFs at proper proportions together with an efficient delivery system are necessary to enhance the biological function of this GF-related method, so PRP/PLEL was developed in this study to meet the aforementioned needs.

Hydrogels have the feature of uniform interconnected micropores, which endows them with a high water retention capacity that not only facilitates mass exchanges, but also prevents exudates build-up on wound sites by promoting drainage. Moreover, for PRP/PLEL, after platelets in PRP release various GFs through degranulation of α granules, these secreted factors can be immobilized in the aforementioned biomimetic structures of PLEL hydrogel through physicochemical interactions. Then the immobilized GFs are slowly released in correspondence with PLEL degradation over a prolonged time period (Figure 4). In addition, PRP/PLEL can be applied by means of a minimally invasive injection, undergoing an instant sol−gel phase transition, which is very important for in vivo application because it markedly reduces operation waiting time. All these features make PRP/PLEL to be a promising candidate for new types of wound dressings in the clinic.

In skin tissue engineering, provision of nutrients and removal of wastes early after wounding are dependent on the permeability of exotic dressings. Afterwards, intrinsic functional vascular networks derived from the neovascularization process develop into the key factor [34], because new vessels can supply oxygen and nutrients as well as circulate necessary cells and humoral factors to the wound site. Neovascularization consists of vasculogenesis and angiogenesis. Vasculogenesis is the initial stage, characterized by mobilization of endothelial progenitor cells (EPCs) from bone marrow and subsequent differentiation into endothelial cells (ECs) to form primitive leaky vessels. Immunohistochemical staining for CD31, a transmembrane protein of ECs, can be used to evaluate these newly formed blood vessels [35]. Angiogenesis involves sprouting of vessels and their following stabilization aided by surrounding smooth muscle cells, which means maturation of vessels occurs in this stage. Plasma protein α-SMA in particular could be applied to indicate these smooth muscle cells [33]. Thus, immunofluorescence co-staining of CD31 and α-SMA was employed to display the mature blood vessels [34]. Immunohistochemistry and immunofluorescence results in this study (Figure 9) showed that up to 14 days, PRP/PLEL effectively raised the number of newly formed as well as mature blood vessels, contributing to accelerated granulation tissue and collagen formation, which are pivotal processes in wound healing. To conclude, PLEL hydrogel immobilized PRP GFs can exhibit a sustained release for at least 14 days with their biological activities retained, which in turn benefits skin wound healing a lot.

Reepithelialization is very important for skin wound healing because epithelium can protect the host from infections. It is reported that epithelialization can be accelerated if the wound is kept moist while dry scab retards it [20,36]. In this study, when compared to control wounds, PRP/PLEL-treated wounds showed significantly better wound closure and thicker epidermis (Figure 7 and Figure 8A). The reason was that PRP/PLEL-treated wounds kept hydrated throughout the whole healing period (Figure 7A), so keratinocytes could crawl more quickly over this wetted wound surface than underneath a scab. Additionally, PRP GFs promoted the formation of blood vessels (Figure 9), which further facilitated keratinocytes’ migration, survival and proliferation and finally led to a better reepithelialization. 

Another important biological process beneficial to wound healing is the replacement of granulation tissue by fibrous tissue [37]. According to the results of Masson’s trichrome staining (Figure 8B), wounds treated with PRP/PLEL displayed the most densely packed collagen fibrils among all the groups, and these fibers were in good alignment, resembling those of normal skin. This positive effect provided further confirmation of our hypothesis that PLEL hydrogel indeed possesses sustained-release capabilities for PRP GFs and facilitates ECM remodeling.

There are several limitations to our study. First, the majority of the PRP/PLEL-treated wounds appeared to be healed within 14 days post surgery, thus whether PRP GFs can exert their biological effects in PLEL hydrogel for more than 14 days to heal some chronic skin wounds needs further in vitro and in vivo study for supplementary evidences; additionally, contraction is an unwanted process during wound healing in clinics as it causes significant functional or aesthetic consequences. However, in this study, contraction actually played an important role in wound closure. In our future research, we will adopt novel methods to limit wound contraction and investigate the effects of PRP GFs on reepithlialization and wound closure. Second, it was reported that PRP contains a relatively high amount of GFs such as PDGF-BB, stromal cell-derived factor-1 (SDF-1α) and insulin-like growth factors-1 (IGF-1), while lacking vascular endothelial growth factor (VEGF) and basic fibroblast growth factor (bFGF) [14]. Nevertheless, VEGF is a principle stimulatory factor for angiogenesis [38] and bFGF is a chemoattractant to smooth muscle cells [39]. That is to say, both VEGF and bFGF have a vital impact on the wound-healing process. More in-depth studies should be performed to determine if there are synergistic effects by incorporating PRP together with exogenous VEGF or bFGF in PLEL hydrogel, leading to better wound-healing results. Third, more and more studies have shown that gelatin or chitosan-based hydrogels can also act as excellent drug-delivery systems [33,37]; comparative studies are therefore needed to decide which is the most effective drug vehicle for clinical use.

## 4. Materials and Methods

### 4.1. Materials

d,l-lactide, PEG and stannous octoate (Sn(Oct)_2_) were purchased from Sigma-Aldrich (St. Louis, MO, USA). Other chemicals and solvents in reagent grade were all purchased from GuoYao Regents Company (Shanghai, China) except where otherwise indicated.

### 4.2. Synthesis, Characterization of PLEL Triblock Copolymers

Detailed methods of PLEL triblock copolymer synthesis and purification had been described previously [20,40]. Briefly, calculated amounts of d,l-lactide and PEG were placed in a dried reaction tube, and Sn(Oct)_2_ was added as catalyst. The tube was heated at 100 °C in vacuum for 1 h to eliminate the trace amount of water from reactants. Then, the reaction process was carried out at 140 °C for 12 h with argon protection. Afterwards, unreacted homopolymers were removed from the as-obtained products through the following order: dissolving in acetone, precipitating using large amounts of water, filtering, and washing by hot water. Finally, the purified products were dried in a vacuum oven at 60 °C for 3 days to gain colorless, transparent PLEL triblock copolymers. ^1^H NMR analysis and FTIR spectroscopy were used to characterize the copolymers according to previous protocols. Briefly, ^1^H NMR spectra (with samples dissolved in CDCl_3_) was performed at room temperature with a spectrometer at 400 MHz, giving the chemical shifts in ppm by using tetramethylsilane (TMS) as an internal reference. For FTIR assay, copolymer powders were dissolved in acetone, mixed with KBr (1:100) and pressed into a disk, analysis was then conducted on an FTIR spectrometer (Nicolet) from 500 to 4000 cm^−1^.

### 4.3. Sol–Gel–Precipitation Phase Transition Behavior Study of PLEL Hydrogel

Firstly, PLEL hydrogels at different concentrations were prepared. The sol–gel–precipitation phase transition behavior of the PLEL hydrogel was investigated using tube-inverting method and dynamic rheological assay [16,20,41]. Briefly, PLEL hydrogel samples were loaded into tightly screw-capped centrifuge tube, and then heated from 10 °C at a rate of 1 °C/min, to the temperature when sol–gel and gel-precipitation phase transition occurred. This transition process was visualized by inverting the tubes; sol and gel conditions were defined as “flowing liquid sol” and “no flowing solid gel” in 1 min, respectively. 

Then, rheological study of the PLEL hydrogel (25 wt %) was carried out by using an HAAKE Rheostress 6000 rheometer (Thermo scientific, Waltham, MA, USA) with parallel plates. Sample was firstly placed between parallel plates with diameter of 20 mm and a gap of 1 mm, then a thin layer of low-viscosity silicone oil was carefully overlaid to minimize solvent evaporation. Heating rate of the temperature sweep experiment was set as 1 °C/min. *G*’ and *G*” were measured as functions of temperature. Data were collected using a controlled stress of 4.0 dyn/cm^2^ and a frequency of 1.0 Hz.

### 4.4. In Vitro Cytotoxicity Tests

Murine L929 cells and EaHy926 were used to assess cytotoxicity of PELA hydrogel and its extracts by Cell Counting Kit-8 (CCK-8; Dojindo, Kumamoto, Japan). Cells were cultured in Dulbecco’s modified Eagle’s medium (DMEM, Gibco, Carlsbad, CA, USA) containing 10% fetal bovine serum (FBS; Gibco, Carlsbad, CA, USA), supplemented with 50 U/mL penicillin and 50 U/mL streptomycin at 37 °C in a humidified atmosphere containing 5% CO_2_. PLEL hydrogel was firstly extracted using DMEM with 10% FBS for 24 h, after which sequential dilutions of the stock solution were carried out to obtain leachates in series concentrations. Cells were detached using 0.25% trypsin–EDTA (Invitrogen, Rockford, IL, USA) and distributed in a 96-well plate at a density of 3 × 10^4^ cells/well and incubated for 24 h. Fresh medium with different concentrations of PLEL hydrogels or their leachates were added and incubated for another 48 h. Subsequently, CCK-8 was applied according to manufacturer’s instructions. The cytotoxicity was expressed as the relative viability (%), with no hydrogel or hydrogel leachate in culture media as 100%.

### 4.5. PRP and PRP/PLEL Composite Preparation

PRP was prepared by modified traditional two-step centrifugation method as previously described [42]. Sprague-Dawley rats (2 months old; 250 ± 12 g) were used in this part; these isogenic individuals were both donors and recipients of PRP to simulate autologous implantation. Briefly, animal blood samples (10 mL/rat) were collected into centrifuge tubes before an acid citrate dextrose A solution was added for anticoagulation. The mixture was initially centrifuged at 2000 rpm for 10 min, superstratum of yellow plasma was removed to another tube and subsequently centrifuged at 2200 rpm for 10 min. After centrifugation, supernatant plasma was discarded and the remaining approximately 0.8 mL plasma per tube with precipitated platelets was designated PRP. Then, PRP was simply incorporated into PLEL hydrogel by aspirating using a pipette to form a homogeneous PRP/PLEL composite with a final PRP concentration of 10% (*v*/*v*). The composite was transferred into 1 mL syringes attached with 27-gauze needles for further usage.

### 4.6. Release Kinetics of PDGF-BB from PRP and PRP/PLEL

The kinetics of PDGF-BB release from PPR alone and PRP/PLEL were determined by ELISA at set time points. Briefly, samples of PRP, PRP/PLEL and standards were added to a 96-well plate and, after gelation, SBF was added. At predetermined time points, the supernatant was collected and replaced with fresh SBF in the same volume. The released PDGF-BB was quantified using an ELISA kit according to the manufacturer’s instructions. The PDGF-BB concentration was determined by correlation with a standard curve.

### 4.7. EaHy926 Migration and Tube-Formation Assays

The effect of PRP GFs on EaHy926 migration was assessed by transwell assay [34]. In brief, 5 × 10^4^ cells were seeded in the upper chamber of a 24-well transwell plate (pore size = 5 μm; Corning, Inc., Corning, NY, USA). Then 600 μL medium containing PLEL, PRP and PRP/PLEL (with the latter two groups contained the same amounts of PRP) were separately added to the lower chamber. After incubation for 24 h, cells on the upper surface of the filter membranes were wiped off with cotton swab, and those that migrated to the lower surface were fixed with 4% paraformaldehyde, stained with 0.5% crystal violet for 10 min, and counted using an inverted microscope.

For tube-formation assays, confluent EaHy926 were pretreated with serum-free medium for 24 h and then seeded at the density of 3 × 10^4^ cells/well in a 24-well plate precoated with 500 μL Matrigel per well, after which the same medium as the above migration assay was added. After a 24 h wait after cell seeding, tube-formation images were taken and the number of complete capillaries connecting individual points of the polygonal structures was counted using an inverted microscope.

### 4.8. Cell Proliferation Analysis

The bioactivity of GFs released from delivery vehicle was determined by assessment of EaHy926 proliferation. Cells were seeded in a 24-well transwell plate with a 3 μm pore polyester membrane at a density of 4 × 10^3^ cells/well and cultured in serum-free medium for 24 h to permit adhesion. Then pre-gelated PLEL, PRP and PRP/PLEL in discoid shape with the same PRP amount was applied above the cultured cells. The culture medium was changed every 4 days. Cell proliferation was assessed by counting the cell number every 4 for 14 days using a hemocytometer.

### 4.9. Skin Wound Treatment

All animal procedures were approved by the Animal Research Committees of Shanghai Six People’s Hospital. A total of 48 Sprague-Dawley rats (2 months old; 250 ± 12 g) were divided into 4 groups of 12 rats. All rats were housed singly at controlled temperature of 20–22 °C, relative humidity of 50%–60% under natural light/dark conditions, and received food and water ad libitum. Prior to surgery, rats were anesthetized with an intraperitoneal injection of 3% pentobarbital sodium solution (Sigma) at a dosage of 1.0 mL/kg. Dorsal hair was first shaved using a shaving machine, then a 10% povidone-iodine and 70% alcohol solution were used for disinfection in three iterations, and full-thickness skin wounds of 2 × 2 cm^2^ were created on each rat by excising the dorsum down to the panniculus carnosus muscle layer with a no. 10 sterile surgical scalpel. PLEL, PRP and PRP/PLEL prefilled in syringes were immediately squirted out and spread evenly onto the established wounds. Owing to increased temperature in the rats’ skin, gelation occurred within 2 min, conforming to the shape of the defects. Wounds were then covered by polyurethane film (Tegaderm, 3M Healthcare, St. Paul, MN, USA), and the trunks of the rat bodies were fixed with elastic adhesive bandage to prevent the rats from removing the dressings. For comparison, wounds treated with polyurethane film and bandage alone served as control. After surgery, rats were kept in separate cages and observed each day to guarantee that the wound dressings were intact. Carprofen (5 mg/kg body weight) was injected subcutaneously as a painkiller once a day for 3 days post surgery.

### 4.10. Wound Analysis

Wounds were grossly examined and digital images were taken by placing a ruler along the side at 0, 5, 10 and 14 days post surgery, wound areas were measured using an image analysis software (NIH Image, Rockville, MD, USA). The above measurements were then used to calculate the percent of wound closure as follows: ((Area of original wound − Area of actual wound)/Area of original wound) × 100%.

### 4.11. Assessment of Reepithelialization, Collagen Deposition and Angiogenesis

Histological evaluation was performed according to previous reports mainly for general histopathological and collagen examination after 14 days [43,44]. Tissue specimens composed of the entire wound and 0.5 cm adjacent normal skin were fixed in 10% buffered formalin solution, embedded in paraffin, and sectioned perpendicularly to the surface of the wound. 4 μm-thick sections were stained using HE reagent and Masson’s trichrome, examined using light microscopy (Olympus BX 45, Olympus, Hamburg, Germany). Immunohistochemical staining for CD31 (1:200, Abcam, Cambridge, UK) and immunofluorescence staining for CD31 + α-SMA (1:50, Abcam, Cambridge, UK) of the sections were conducted to evaluate vascular system formation [34]. Corresponding number of blood vessels were counted in a blind fashion by two pathologists.

### 4.12. Statistical Analysis

The data were expressed as mean ± SD. One-way analysis of variance (ANOVA) or a Student’s *t*-test was used to determine the level of significance, and *p* values less than 0.05 were considered statistically significant. Data analysis was performed using SPSS 10.0 (SPSS, Chicago, IL, USA).

## 5. Conclusions

In this study, we successfully prepared an injectable thermosensitive in situ gel-formation poly(d,l-lactide)-poly(ethylene glycol)-poly(d,l-lactide) (PLEL) hydrogel, in which platelet-rich plasma (PRP) could be incorporated simply and homogeneously, and this combination makes sustained release of PRP growth factors (GFs) possible. In vitro, the PLEL hydrogel was demonstrated nontoxic to vascular endothelial cells and fibroblasts; furthermore, PRP/PLEL could stimulate EaHy926 proliferation, migration and tube formation. In vivo studies showed that the PRP/PLEL composite facilitates angiogenesis and skin wound healing. Taken together, these results demonstrated that PRP containing potent angiogenic GFs together with PLEL hydrogel were effective in restoring the completeness of severe skin defects, and PRP/PLEL may possibly be used as a therapeutic strategy in the management of patients with full-thickness skin wounds.
